# Hexavalent chromium at the crossroads of science, environment and public health

**DOI:** 10.1039/d5ra03104d

**Published:** 2025-06-25

**Authors:** Yaroslav Zhigalenok, Aigerim Tazhibayeva, Saule Kokhmetova, Alena Starodubtseva, Tatyana Kan, Dana Isbergenova, Fyodor Malchik

**Affiliations:** a Al-Farabi Kazakh National University Almaty 050040 Kazakhstan tornatore@mail.ru

## Abstract

Hexavalent chromium (Cr(vi)) contamination represents one of the most persistent and complex environmental challenges of our time. This comprehensive review synthesizes current knowledge across toxicology, environmental geochemistry, analytical chemistry, and remediation technologies to reveal fundamental disconnects between scientific understanding and practical solutions. While research has elucidated molecular mechanisms of Cr(vi) toxicity with remarkable precision – from cellular entry through oxidative damage cascades to genomic instability – this knowledge has not translated into proportionally effective environmental remediation strategies. The analysis reveals that chromium contamination is more complex and persistent than traditionally acknowledged. The reversible nature of chromium redox transformations creates dynamic contamination cycles that resist conventional treatment approaches. Emerging evidence challenges the traditional safe Cr(iii) *versus* toxic Cr(vi) paradigm, suggesting all chromium forms may pose health risks under certain conditions. Critical assessment of current remediation technologies demonstrates that while laboratory studies consistently report high removal efficiencies, these approaches fail to address the vast scale of existing environmental contamination. Most critically, conventional methods focus on transferring chromium between phases rather than implementing circular economy principles that enable recovery and reuse of this valuable element. The review concludes that to address the chromium crisis, it is necessary to move beyond conventional wastewater treatment and adopt prevention-focused strategies that emphasize circular economy principles. Future solutions must prioritize contamination prevention, closed-loop industrial systems, and long-term management rather than pursuit of complete remediation. Only through such realistic assessment and integrated action can we hope to minimize the ongoing impacts of this persistent environmental challenge.

## Abstract 2

A critical review of the Cr(vi) lifecycle, from geogenic and industrial sources and molecular toxicity mechanisms to the practical challenges of modern detection and remediation technologies.

## Graphical abstract

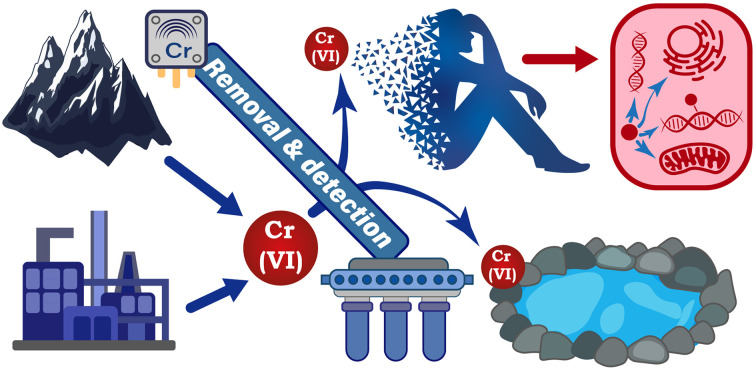

## 1 Introduction

Industrial progress has relied on chemical compounds, many later found to pose serious environmental and health threats. Hexavalent chromium (Cr(vi)), a highly toxic and carcinogenic substance, is one such legacy, and its presence in ecosystems is now a global problem.^1^ The widespread use of chromium compounds in industries such as electroplating, leather tanning, pigment production, metallurgy, wood preservation, and many others^2–4^ has led to massive discharges of chromium-containing wastewater over decades. As a result, Cr(vi) is a common and dangerous pollutant in industrial effluents, soils, and alarmingly, in drinking water sources worldwide.^5–7^

Hexavalent chromium (Cr(vi)) is classified by the International Agency for Research on Cancer (IARC) as a Group 1 carcinogen, meaning a substance with proven ability to cause cancer in humans.^8,9^ In the United States, it consistently ranks among the top twenty most dangerous pollutants at monitored sites.^8^ Cr(vi) is highly mobile in water and easily penetrates cell membranes of living organisms.^10,11^ Once inside the cell, Cr(vi) initiates destructive processes: it causes powerful oxidative stress, directly damages DNA and proteins,^12,13^ disrupts cellular metabolism and energy production,^14^ causing many pathologies, including mutations, cell death, and cancer.^13,15^ Acute exposure to Cr(vi) can cause severe damage to internal organs – kidneys, liver, respiratory system,^6^ while chronic exposure is associated with increased risk of developing serious diseases.^13,16^

Cr(vi) enters the environment not only from industrial sources. Significant concentrations are also found in natural conditions, released during the weathering of chromium-containing rocks, especially under specific hydrogeochemical conditions.^5,17,18^ This natural origin complicates water quality control, especially in areas with relevant geology. Therefore, Cr(vi) contamination is a complex problem with serious consequences for public health and ecosystems.

In response to this threat, the global scientific community is making significant efforts to develop methods for removing Cr(vi) from wastewater and contaminated natural environments. The spectrum of proposed technologies is extremely broad and includes physicochemical approaches such as adsorption on various materials,^19^ ion exchange methods,^20^ membrane filtration,^21^ chemical reduction and precipitation,^22^ electrochemical methods,^23^ and photocatalysis,^24^ as well as biological methods using the ability of microorganisms^25^ and plants^26^ to absorb or transform. Parallel efforts have focused on developing advanced detection and monitoring systems, including portable colorimetric sensors,^27^ smartphone-based analytical devices,^28^ fluorescent probes,^29^ and real-time monitoring platforms^30^ that enable rapid field assessment of contamination levels. However, despite the diversity of approaches and the constant emergence of new developments, effective, economically viable, and environmentally safe solutions to the Cr(vi) problem on an industrial scale still remain an ongoing challenge.

The hexavalent chromium (Cr(vi)) problem has been the subject of numerous studies, reflected in an extensive body of scientific literature. A multitude of review articles focus on specific, albeit important, aspects, such as the mechanisms of its toxicity,^10,31,32^ its geochemistry,^33^ detection methods^34,35^ or specific cleanup technologies.^8,36–39^ The problem's complexity, stemming from the vast scale of environmental contamination, also demands a broader, interdisciplinary perspective. However, comprehensive reviews that bridge all fields are exceptionally rare. To our knowledge, at least one comparable study that covers sources, toxicity, and remediation together.^40^ While valuable, that review also maintains a specific focus on green bioremediation technologies, rather than providing a critical analysis of the practical barriers facing the full spectrum of cleanup methods.

This review provides a synthetic and critical perspective on the systemic nature of hexavalent chromium (Cr(vi)) contamination. It connects the fundamental mechanisms of Cr(vi) toxicity with its complex environmental behavior to substantiate public health risks. The review also presents an overview of modern detection methods, which are crucial for risk management. A key focus is the critical assessment of existing cleanup technologies from the standpoint of their practical effectiveness. By integrating findings from toxicology, geochemistry, analytical chemistry, and materials science, this work aims to foster a holistic understanding of the threat and identify promising directions for future interdisciplinary research.

This review is based on a systematic analysis of scientific literature from the Scopus, ScienceDirect, and Web of Science databases. The selection process prioritized peer-reviewed English-language publications and was conducted in two stages. Foundational works on chromium's fundamental toxicology and geochemistry, including seminal papers from the 1980s and 1990s, were included to provide essential context. The core analysis, however, is built upon current research from the last 5–7 years, focusing specifically on recent developments in detection, adsorption, and remediation technologies. All selected literature underwent a multi-stage screening, first by title and abstract, then by a full-text evaluation for available publications.

## 2 Biological damage of hexavalent chromium

Hexavalent chromium (Cr(vi)), found everywhere from industry and natural processes, is a toxicant causing deep concern in the global community due to its widespread presence in the environment and its proven detrimental effects on living organisms.^41^ Its danger contrasts sharply with Cr(iii), whose biological role is still debated – formerly considered essential, but now with emerging data on its toxicity and ability to cause genomic instability under certain conditions.^42^ Unlike it, the hexavalent form of chromium is unequivocally recognized as a highly toxic substance, mutagen, and potent human carcinogen.^43^ The destructive action of Cr(vi) is not limited to carcinogenesis alone. The complex threat from acute or chronic exposure makes the Cr(vi) problem a priority for modern toxicology and public health. This chapter is devoted to the critical analysis of the biological damage inflicted by this element.

### 2.1. Clinical presentation of chromium intoxication in humans

Human exposure to hexavalent chromium (Cr(vi)) leads to a range of pathological conditions, which include severe effects from acute poisoning as well as chronic diseases, such as cancer.

Acute poisoning with high doses of hexavalent chromium (Cr(vi)), typically through the ingestion of concentrated solutions, causes severe and often irreversible damage. For instance, a documented case reported the death of an electroplating plant worker who accidently drank a production solution containing chromium.^44^ The clinical progression begins with initial symptoms like nausea and severe abdominal pain, followed by a rapid onset of multi-organ failure. The kidneys are a primary target organ in acute chromium ingestion, but the systemic toxicity extends to other vital systems, including the liver, gastrointestinal tract, and the cardiovascular and immune systems. Toxicological analysis in such cases confirms the rapid absorption and distribution of chromium throughout the body's tissues and organs.

While acute Cr(vi) poisoning is rare, chronic exposure to lower doses poses a more widespread and subtle threat, especially for workers in certain industries and populations living in environmentally compromised areas.^41,45,46^ The most ominous long-term consequence of chronic Cr(vi) exposure is the development of malignant neoplasms. Hexavalent chromium compounds are unequivocally recognized by IARC as carcinogens to humans, primarily based on the indisputable link with lung cancer found in workers engaged in chromate production, chromate pigments, electroplating, and stainless steel welding.^47^ Data on the association between oral or occupational Cr(vi) exposure and stomach cancer remain ambiguous: while one large meta-analysis found an increased risk,^48^ another did not confirm a statistically significant increase in the risk of stomach cancer or other GI tract cancers from occupational exposure,^49^ which may indicate the complexity of assessing this risk or dependence on the specifics of cohorts and exposure levels. Recent *in vitro* and *in vivo* studies demonstrate Cr(vi)'s ability to stimulate proliferation and invasion of prostate cancer cells, which, coupled with data on elevated chromium levels in serum of such patients, indicates its potential role in the progression of this disease, although this issue requires further detailed study.^50^ Beyond oncological diseases, long-term Cr(vi) exposure causes a wide spectrum of non-carcinogenic toxic effects affecting multiple organs and systems.

The respiratory system, being the main entry route for Cr(vi) during occupational exposure, suffers not only from the risk of cancer development. Chronic inhalation of Cr(vi) compounds leads to irritation of mucous membranes, chronic inflammatory diseases, development or exacerbation of bronchial asthma.^41^ Studies also show direct structural damage to lung tissue, including alterations at the alveolar level and disruption of mitochondrial function in epithelial cells,^13,51^ which inevitably reduces the efficiency of gas exchange between air and blood and may contribute to the development of chronic respiratory failure.

The skin, as the first barrier against toxicants during contact exposure, is also subject to its aggressive influence. This manifests as characteristic contact dermatitis, allergic reactions, and the formation of difficult-to-heal “chrome ulcers”.^52^ At the cellular level, Cr(vi) has been shown to disrupt the skin's barrier function by damaging intercellular junctions and inducing apoptosis of keratinocytes^53^ – that is, death of the main epidermal cells. The reduction in the number of these cells weakens the skin barrier: it thins, loses its integrity, which opens the way for infections and makes the skin more susceptible to further chemical damage.

The kidneys, as a vital organ of the excretory system, are particularly vulnerable to Cr(vi) exposure, especially in acute poisoning, but also with chronic accumulation. Nephrotoxicity develops, characterized by damage to the renal tubular epithelium, which can lead to impaired filtration and reabsorption function of the kidneys.^54^ As a result, the kidneys lose their ability to effectively cleanse the blood of metabolic waste and toxins, as well as maintain the necessary water-salt balance in the body. This damage often results in proteinuria, the leakage of proteins into the urine, which are normally retained in the blood during healthy kidney function.^55^ In severe cases, this progresses to chronic renal failure requiring dialysis or transplantation.

The liver, as the center of metabolism and detoxification, also serves as a target for Cr(vi). Hepatotoxic effects manifest in disruption of metabolic functions and degenerative changes in hepatocytes (the main working cells of the liver). With prolonged exposure, activation of hepatic stellate cells is described, triggering the development of liver fibrosis.^56^ Fibrosis is a pathological process of replacing normal, functional liver tissue with coarse connective (scar) tissue. As fibrosis progresses, the liver loses its functions, which can lead to the development of cirrhosis – a severe, irreversible condition.

Also of concern is the reproductive toxicity of Cr(vi), which threatens fertility and offspring health. In women, severe consequences such as follicular atresia^57^ – that is, premature degeneration and death of follicles containing eggs, which directly leads to depletion of the egg supply (ovarian reserve) and development of infertility – disruption of steroidogenesis and delayed puberty are described, with epigenetic changes in ovarian tissue possibly underlying these disorders.^57^ The male reproductive system is no less vulnerable: Cr(vi) causes testicular tissue damage, leads to decreased testosterone levels, disrupts lipid metabolism processes and autophagy in the testes,^58^ and also exhibits toxicity toward spermatogonial stem cells.^59^ These cells are the progenitors of all sperm and ensure their continuous production throughout a man's life, so their damage or death can lead to serious and prolonged, up to irreversible, impairment of male fertility. Reproductive dysfunction may also be related to the toxic effect of Cr(vi) on the pituitary gland, a regulator of the endocrine system.^60^

**Fig. 1:**
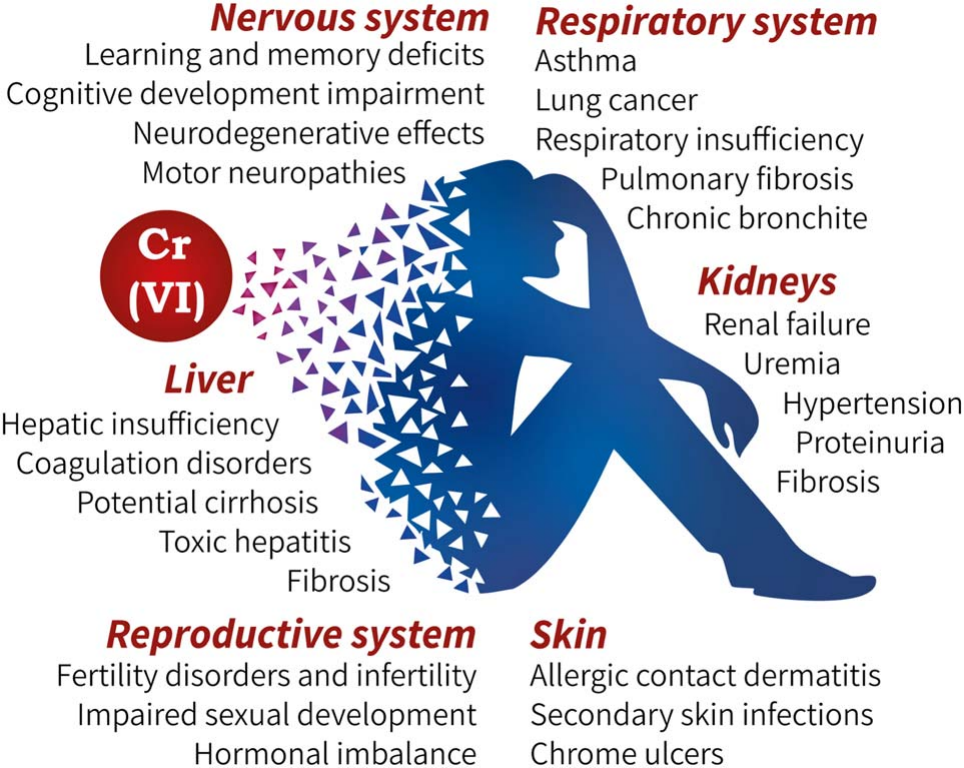
Main clinical manifestations of possible consequences of toxic exposure to Cr(vi) on human organs and systems.

Finally, increasing evidence points to the neurotoxic potential of Cr(vi), although this area still requires active study. Animal model studies demonstrate behavioral, learning, and memory impairments with chronic Cr(vi) consumption in drinking water even at concentrations considered relatively safe.^61^ Review data suggest a possible link between Cr(vi) exposure and cognitive development disorders in children, deterioration of smell and social memory, and also suggest its potential contribution to the development of neurodegenerative processes and motor neuropathies in adults,^31^ which inevitably affects quality of life, learning ability, and social adaptation of those affected. The entire spectrum of described pathological effects of Cr(vi) on the main systems of the human body is summarized in Fig. 1.

The diverse pathological effects of hexavalent chromium (Cr(vi)), such as lung cancer, kidney failure, infertility, and memory loss, share a common underlying mechanism: the destruction of specialized cells within various organ systems. Cr(vi) targets multiple cell types, including respiratory epithelium, liver hepatocytes, kidney nephrons, as well as skin, reproductive, and nerve cells. The death or dysfunction of these functional cells leads directly to the failure of their respective systems, such as respiration, detoxification, excretion, reproduction, and cognitive functions. Thus, the clinical presentation of Cr(vi) toxicity reflects a systemic failure at the cellular level.

### 2.2. Molecular mechanisms of Cr(vi) induced cell damage

The primary factor determining the toxicological activity of Cr(vi) is its ability to easily overcome the cellular barrier – the plasma membrane. In physiological conditions, Cr(vi) exists predominantly as a tetrahedral chromate anion (CrO_4_^2−^), which in structure and charge resembles vital anions for the cell – phosphate (PO_4_^3−^) and sulfate (SO_4_^2−^). Due to this structural similarity, Cr(vi) effectively uses cellular transport systems and is actively transported into the cell by proteins that carry these anions, such as chloride intracellular channels (CLIC).^62^ This mechanism ensures rapid entry of the toxic compound into the cytoplasm. This represents a cardinal difference from trivalent chromium (Cr(iii)), which exists as a cation and for which cell membranes are practically impermeable.^63^

Once inside the cytoplasm, Cr(vi) undergoes a multi-stage reduction to lower oxidation states: Cr(vi) → Cr(v) → Cr(iv), and finally to the relatively stable form, Cr(iii).^64,65^ This process is essentially a toxic activation of chromium inside the cell. This reduction occurs through two main pathways. The first is a non-enzymatic process involving low molecular weight reducing agents like ascorbic acid (vitamin C)^66^ and glutathione (GSH).^63,67,68^ The second pathway utilizes enzymatic systems, such as cytochrome P450 reductase and glutathione reductase.^65,68^ The main toxicity of Cr(vi) is caused not by the final reduction product (Cr(iii)), but by the process itself and the short-lived but extremely reactive intermediate forms – Cr(v) and Cr(iv) ions.^64,65^

The intracellular reduction of Cr(vi) inevitably triggers powerful oxidative stress, a state where the cell's ability to neutralize aggressive oxidants is overwhelmed. Unstable chromium ions (Cr(v), Cr(iv)) actively react with molecular oxygen, leading to the massive generation of reactive oxygen species. These damaging particles include the superoxide radical (O_2_^−^), hydrogen peroxide (H_2_O_2_), and the extremely aggressive hydroxyl radical (OH˙).^43,69,70^ The cell's antioxidant defense systems, which normally handle ROS, are quickly overloaded. Key protective enzymes are depleted or suppressed, and the cell's primary non-enzymatic antioxidant, glutathione (GSH), is critically depleted.^70–72^ As a result, ROS can freely attack vital cellular components like lipids, which form the structure of all cellular membranes, disrupting their integrity and function.

The attack of ROS on lipids, which form the structural basis of cellular and intracellular membranes, leads to their oxidation (formation of lipid oxidation product – LOP) – a chain reaction of oxidation by free radicals, which disrupts the structure and fluidity of membranes, their barrier function and integrity.^73^ Damage to the membranes of mitochondria, lysosomes, and the plasma membrane itself opens the path to further cellular dysfunction and death.

One of the main targets of Cr(vi), which determines its mutagenic and carcinogenic potential, is the cell's genetic material – DNA. This damage to the genome, known as genotoxicity, is a multifactorial process resulting from both oxidative stress and the direct interaction of chromium ions with DNA.^74,75^ This assault on the genome leads to various types of damage, including Cr-DNA adducts (where chromium directly binds to DNA), DNA-protein crosslinks, and breaks in the DNA chains.^12,76,77^

If this damage is not properly repaired by the cell's systems, or if those systems are overloaded, the consequences for the genome are severe. These include point mutations (changes in the DNA sequence), large-scale chromosomal aberrations (such as breaks, deletions, and translocations of chromosome parts), and changes in the total number of chromosomes (a condition known as aneuploidy).^74,78,79^ All of this leads to a state of genomic instability – an increased frequency of genetic changes, which is a fundamental characteristic of cancer cells and a major driver of their development.^80^

However, the genotoxicity of Cr(vi) is not limited to direct DNA damage. There is growing evidence for the important role of epigenetic mechanisms – heritable changes in gene expression that are not caused by alterations in the DNA sequence itself. Cr(vi) has been shown to alter DNA methylation patterns, affect modifications of histones (the key proteins that package DNA),^9,57^ cause instability in ribosomal DNA (rDNA),^15^ and disrupt the expression of microRNAs (small regulatory molecules that control gene activity).^81^ Epigenetic marks normally function as a precise system for “switching” genes on and off at the right time. When Cr(vi) disrupts this system, genes that stimulate cell growth (proto-oncogenes) can become permanently “switched on”, while genes that should stop uncontrolled division or trigger DNA repair (tumor suppressor genes) can be erroneously “switched off”.

Cr(vi) not only damages DNA but also disables the cell's repair systems. A key repair pathway for severe DNA damage, known as homologous recombination, has been shown to be inhibited by prolonged Cr(vi) exposure.^12,82^ This creates a double-hit scenario: the cell sustains more genetic damage while its ability to fix it is compromised.

The accumulation of these molecular damages – oxidative chaos and genomic instability – inevitably leads to a profound dysfunction of cellular systems. Beyond DNA, another key target is the mitochondria, the cell's energy stations. Cr(vi) damages their membranes, disrupts their respiratory processes, and reduces the synthesis of ATP (the cell's main energy currency). As a result, the cell's metabolism shifts toward anaerobic glycolysis, a much less efficient way of producing energy.^13,14,69^

The impact of Cr(vi) also extends to the endoplasmic reticulum (ER), the cell's factory for producing and folding proteins. The accumulation of defective proteins inside the ER causes a condition known as ER stress. The cell responds with a special program called the unfolded protein response (UPR) to restore order or, if the damage is too severe, to trigger cell death. The effect of Cr(vi) on ER stress is dose-dependent: low concentrations induce moderate stress, while high concentrations can suppress the response.^83^ The transcription factor ATF4 plays a key role here, altering the cell's metabolism and promoting survival at low doses of Cr(vi), while its levels decrease at high doses.^84^

Cell cycle regulation is also thrown into disarray. In response to DNA damage, checkpoints may be activated (for example, in the G2/M phases) to stop cell division and allow time for repairs. However, with strong or prolonged exposure, these protective mechanisms can be overridden or damaged, contributing further to the accumulation of mutations and chromosomal instability.^12^ Additionally, Cr(vi) interferes with numerous intracellular signaling pathways that control critical processes. These include pathways regulating cell growth and differentiation, such as Hedgehog^85^ and EMT;^50^ pathways crucial for cell survival that manage stress and apoptosis, like Akt,^69^ ATR,^66^ and Nrf2;^43^ and those that control cellular metabolism, such as AMPK^69^ and HIF1α.^86^

**Fig. 2:**
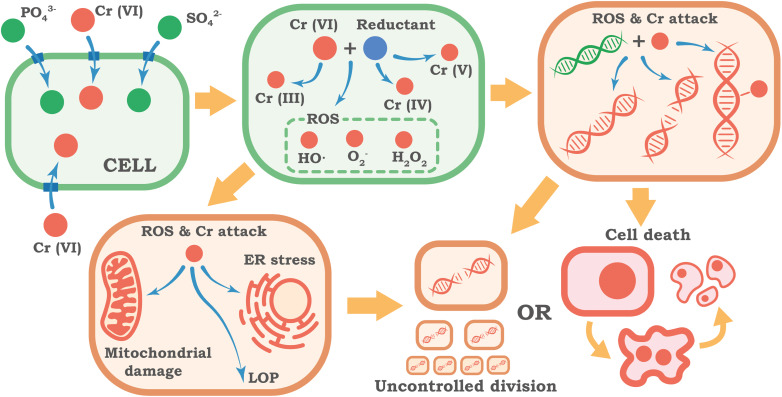
Molecular mechanisms of Cr(vi) cellular toxicity.

Ultimately, when the level of damage exceeds the cell's ability to adapt and repair, mechanisms of cell death are triggered. Depending on the context, this may be apoptosis – active, programmed cellular death,^53,60,72,86^ necrosis – passive, uncontrolled cell disintegration,^70,87^ or other forms of regulated death, such as pyroptosis^87^ or death associated with autophagy/mitophagy.^58,59^ A visual summary of the molecular mechanisms of Cr(vi) toxicity considered is presented in Fig. 2.

The mass death of these functionally active cells – be they in the liver, kidneys, lungs, or reproductive system – is the direct cause of the organ-level damage described in Section 2.1. The loss of a significant part of the cell population leads to organ failure, chronic inflammation, and fibrosis. If, however, a cell manages to avoid death despite the accumulated genetic and epigenetic damage, it has a direct predisposition to uncontrolled division and malignant transformation, leading to the development of cancer.

Thus, the toxicity and carcinogenicity of hexavalent chromium are caused by a unique and destructive cascade of events. Its easy entry into the cell, its subsequent toxic activation, the induction of powerful oxidative stress, and the multi-pronged assault on the genetic apparatus (including DNA damage, epigenetic alterations, and suppression of repair) all lead to the disruption of key cellular functions or cell death. These events at the cellular level directly translate into the pathologies observed at the level of the whole organism – organ failure, systemic diseases, and cancer – which explains why Cr(vi) presents such a serious and complex biological hazard.

### 2.3. Controversial issues and uncertainties in chromium toxicology

Despite the extensive body of data on the clinical manifestations and molecular mechanisms of hexavalent chromium toxicity, attempts to provide an assessment of its actual risk to human health and ecosystems face a number of serious obstacles. Existing scientific contradictions, methodological difficulties, and significant knowledge gaps do not allow us to determine with complete certainty the true scale of the threat. Furthermore, data suggests current risk assessments may significantly underestimate the long-term effects of Cr(vi) exposure.

The traditional opposition of “bad” Cr(vi) and “good” Cr(iii) is increasingly being challenged. Historically, Cr(iii) was considered minimally toxic^42,47,88^ and an essential element (though its essentiality is disputed^89^), and its formation from Cr(vi) was seen as detoxification. However, data is accumulating on the significant biological activity of Cr(iii) itself, especially when formed intracellularly or introduced as complexes (such as chromium picolinate^42^). It exhibits geno- and cytotoxicity: it can directly interact with DNA, alter its structure, disrupt transcription factor binding and transcription,^90^ cause DNA damage and chromosomal aberrations,^42,91^ induce oxidative stress and apoptosis,^91^ and significantly affect gene expression profiles.^92^ There is evidence of its ability to contribute to genomic instability,^89^ and some Cr(iii) compounds (picolinate) have shown mutagenicity and links to organ damage.^42^ This raises a fundamental question: if the end product of Cr(vi) reduction inside the cell – Cr(iii) – is not itself completely inert and safe, but can cause long-term negative cellular and genetic effects, how correct is it to consider the reduction process exclusively as detoxification?

A significant divergence in regulatory approaches to chromium highlights a gap between the known toxicological risks of Cr(vi) and the standards in practice. For public drinking water, major agencies like the World Health Organization (WHO) and the Bureau of Indian Standards (BIS) set a limit of 0.05 mg L^−1^ for total chromium,^93,94^ while the U.S. Environmental Protection Agency (US EPA) sets a slightly higher limit of 0.1 mg L^−1^.^95^ This choice to regulate total chromium is largely a pragmatic one. The WHO explains this by noting that “current analytical methods and the variable speciation of chromium in water favour a guideline value for total chromium”. Even though this 0.05 mg L^−1^ value was questioned due to Cr(vi)'s known carcinogenicity, the WHO retained it as a “practical measure… unlikely to give rise to significant risks to health” pending further re-evaluation. In contrast, regulations for occupational settings, where the evidence linking exposure to disease is irrefutable, specifically target the most hazardous form. For instance, the U.S. Occupational Safety and Health Administration (OSHA) mandates a strict permissible exposure limit for airborne hexavalent chromium at 5 μg m^−3^ over an 8-hour workday, a standard designed to protect workers from the well-documented risk of lung cancer from inhalation.^96^ This regulatory split is telling. The existence of a stringent, specific standard for Cr(vi) in the workplace acts as an official acknowledgement of which form poses the greatest danger. Consequently, the use of a broader total chromium standard for drinking water appears to be less a statement of safety and more of a practical compromise, dictated by economic and logistical limitations rather than pure toxicological principles.

Another acute area of uncertainty and fierce scientific and regulatory disputes is the carcinogenic mechanism of action (MOA) of low doses of Cr(vi), particularly relevant for drinking water and air regulation.^97^ The central question of the discussion: does Cr(vi) act through a threshold (non-genotoxic) or non-threshold (genotoxic) mechanism? Arguments for a threshold MOA are often based on data showing cancer in rodents only at very high doses of Cr(vi). These data are interpreted as evidence of a non-mutagenic mechanism of action (MOA), associated, for example, with cytotoxicity and resulting compensatory proliferation of intestinal epithelial cells, rather than with direct DNA damage. Such a mechanism implies the existence of some threshold – a dose below which the risk of cancer development is considered negligibly small. This view is supported by, for example, analyses of the ratio of carcinogenic to genotoxic potency of Cr(vi) upon oral administration, showing its similarity to non-genotoxic agents.^98^ Also, MOA studies for the inhalation route indicate predominantly negative results in *in vivo* mutagenicity tests and the role of non-mutagenic events (tissue damage, inflammation),^99^ while calculations of exposure margins (MOE) for current Cr(vi) levels in air may indicate a low level of risk, questioning standard linear models.^100^ Based on such data, some regulators establish relatively high permissible Cr(vi) concentrations.^97^

However, the approach based on a threshold MOA is contested, particularly by the U.S. Environmental Protection Agency (US EPA), which proceeds from the possibility of a non-threshold, genotoxic action (*i.e.*, the assumption that any, even the smallest, dose carries some additional risk) even at low doses.^97^ Although the direct mutagenicity of Cr(vi) *in vivo* remains a subject of debate,^99^ data on the interaction of chromium compounds with DNA *in vitro*, causing structural changes,^76^ is presented in support of potential genotoxicity. Additionally, *in vitro* studies demonstrate Cr(vi)'s ability to cause genomic instability in the form of aneuploidy (changes in chromosome number) at physiologically relevant concentrations.^101^ The possible contribution of epigenetic mechanisms to carcinogenesis is also considered.^100^ Based on this assumption of a non-threshold, mutagenic risk, the US EPA's Integrated Risk Information System (IRIS) has derived a cancer slope factor (CSF) for Cr(vi). This CSF is used to calculate health-protective target concentrations in drinking water corresponding to specific cancer risk levels, which can be as low as approximately 0.07 μg L^−1^ for Cr(vi).^97^ Such derived values for Cr(vi) are thus 2–3 orders of magnitude lower than the enforceable MCL for total chromium (0.1 mg L^−1^ (ref. 95)) and also significantly stricter than standards established by other bodies based on threshold approaches or total chromium measurements. This large gap between the official, legally enforced limit for total chromium and the much stricter health advisory level for toxic Cr(vi) highlights a key problem for regulators. Additional questions about the safety of existing lenient standards for total chromium are also raised by data on neurological effects in animals at concentrations close to these limits.^61^ This lack of consensus on the MOA for low doses of Cr(vi) and how to regulate its specific forms leads to continued regulatory uncertainty and significant differences in risk assessments for the population.

Serious concern is raised by the chronic release of metal ions, including chromium, from widely used CoCr medical implants (orthopedic, dental).^67,71,101,102^ Unlike external exposure, implants provide constant internal release of ions (Cr, Co, *etc.*) due to wear and corrosion,^103^ leading to their elevated levels in patients.^67,71,102^ Experimental data confirms the biological activity of these ions: *in vitro* they cause cytotoxic,^67^ immune,^71^ and genotoxic^101^ effects (chromosomal damage). Clinically, this may manifest as inflammation and metallosis^103,104^ (the pathophysiology of which is insufficiently studied). Of particular concern is the potential carcinogenicity of cobalt and chromium compounds, discussed both in the context of ion release from medical implants and occupational contact. Although a direct causal relationship in humans remains a subject of debate,^105^ rare cases of aggressive tumors (angiosarcoma,^106^ osteosarcoma^105^) near implants have been described. Additionally, a case of lung fibrosis followed by adenocarcinoma has been recorded in a dental technician after many years of contact with dust from cobalt–chromium alloys during polishing; high levels of these metals were found in the patient's lung tissue.^107^ Nevertheless, the combination of proven *in vitro* toxicity, hypothetical carcinogenic risk, and unstudied long-term effects creates a serious unresolved risk assessment problem. The absence of established safe limits for ions coming from implants underscores this underestimated danger for millions of people.

Furthermore, accurate assessment of the actual impact of Cr(vi) on humans is complicated by existing difficulties in biomonitoring. Although methods exist for determining chromium in various biological media (urine, blood, exhaled air^46,108^), the most common and accessible approaches measure total chromium, not reliably differentiating its valence states – toxic Cr(vi) and less toxic Cr(iii).^46^ Given the relatively rapid reduction of Cr(vi) to Cr(iii) in the body (especially in blood), measuring total chromium (particularly in urine, which is the main marker^46^) is an insufficiently specific indicator for assessing exposure specifically to Cr(vi).

In conclusion, it must be emphasized that despite extensive knowledge about Cr(vi) toxicity, the full scale of its danger likely remains substantially underestimated. The scientific contradictions, risk assessment difficulties, knowledge gaps, and monitoring challenges discussed (summarized in Table 1) create significant uncertainty regarding the full extent of chromium's toxicological danger. This uncertainty underscores the need for a precautionary approach, which requires not only intensifying interdisciplinary research and improving monitoring but also revising regulatory frameworks to prioritize prevention. However, a complete risk assessment cannot rely solely on toxicology. To effectively prevent contamination and protect public health, it is equally crucial to understand how hexavalent chromium enters and behaves within the environment. Therefore, the following section will analyze the sources, migration pathways, and transformation processes that define the environmental cycle of Cr(vi).

**Table 1:** Controversial issues and uncertainties in chromium toxicology

Issue/area	Essence of the problem/uncertainties	Arguments/data	References
Cr(iii) toxicity	Is Cr(iii) truly low-toxic/essential, or can it cause harm?	Traditionally low-toxic; but new data shows DNA/cell damage, especially for complexes (picolinate)	42, 47 and 88–92
Regulatory limits	Do total chromium limits in drinking water protect public health?	Water standards for total Cr set for practicality, contrast with strict Cr(vi)-specific occupational limits, suggesting a regulatory compromise not based purely on toxicity	93–97
Low Cr(vi) dose MOA	Threshold (non-genotoxic) or non-threshold (genotoxic) mechanism?	Arguments for threshold: cancers in rodents only at very high doses. Arguments against/for genotoxic: EPA stance, aneuploidy, potential DNA interaction, neuro-effects at low levels	76 and 97–101
Medical implants	Long-term risks from Cr/Co ion release?	*In vitro* cyto-/genotoxicity; *in vivo* inflammation, metallosis; rare cancer cases (causality debated); no safe limits set	67, 71 and 101–107
Biomonitoring	How to accurately assess Cr(vi) exposure (*vs.* total Cr)?	Urine (total Cr) – non-specific; exhaled breath condensate for (Cr(vi)/Cr(iii)) – promising but needs validation	46 and 108
Carcinogenicity for GI tract	Is Cr(vi) a GI carcinogen?	Conflicting meta-analyses: one suggests risk (stomach), another finds no significant risk	48 and 49

## 3 Hexavalent chromium in the environmental cycle

After detailed examination of the mechanisms of toxic and carcinogenic action of hexavalent chromium on biological systems, the logical next step is to analyze its behavior in the environment. The entry of Cr(vi) into ecosystems is caused by both extensive anthropogenic activities related to its industrial use and waste disposal, and natural geochemical processes leading to the release and transformation of chromium from rocks.

As a result of this dual origin and its inherent physicochemical properties, Cr(vi) is found in various components of geospheres. Its further ecological fate is determined migration, sorption, and oxidation–reduction transformations, which control its mobility, bioavailability, and persistence in the environment. A deep understanding of the sources of Cr(vi) contamination, its biogeochemical cycle, and factors controlling its distribution and accumulation in natural reservoirs (soils, surface and groundwater) is a necessary condition for adequate assessment of environmental risks and development of effective control and remediation strategies, which will be discussed in Chapter 3.

### 3.1. Sources of Cr(vi) contamination. Anthropogenic and geogenic factors

Hexavalent chromium (Cr(vi)) enters the environment from both anthropogenic and geogenic sources, with historical attention primarily focused on anthropogenic sources due to their major contribution to global pollution. Numerous industrial processes lead to emissions and discharges of chromium.^2–4^ Particularly notable are the leather industry, which generates enormous volumes of wastewater and sludges often with high chromium content and potential for Cr(vi) formation,^109,110^ and the mining and metallurgical complex, leaving behind slags^111,112^ and ore processing tailings (COPR) where chromium concentration can reach weight percentages.^113,114^ A critically important aspect is the improper management of these wastes: their direct discharge, storage in unprepared sites, burial together with municipal waste or sewage sludge,^115–117^ as well as the utilization of ash and slag materials from thermal power plants,^118^ transform industrial zones and adjacent territories into powerful and long-term sources of contamination. This abandoned sites^119,120^ and old landfills,^113,116^ can replenish ecosystems with toxic Cr(vi) for decades,^121^ forming extensive and difficult-to-eliminate hotspots of soil and groundwater contamination. The scale and persistence of pollution from these well-known anthropogenic sources underscore the complexity of effectively controlling and neutralizing such sites.

Alongside technogenic pollution, geogenic sources make a substantial, and in some regions dominant, contribution to the presence of Cr(vi) in the environment. Chromium is a natural component of the Earth's crust, but its content sharply increases in specific geological formations, primarily in ultrabasic and basic (mafic) rocks.^17,122–125^ The main mechanism of geogenic chromium entry into water systems includes two stages: first, the slow release of trivalent chromium (Cr(iii)) during the weathering of primary chromium-containing minerals;^5,122^ second, the subsequent oxidation of this relatively inert Cr(iii) to the highly mobile and toxic form Cr(vi). Natural processes such as forest fires on chromium-rich soils can also contribute to its mobilization and potential oxidation.^126^ Intensive weathering and oxidation in favorable geochemical conditions can lead to the formation of significant concentrations of geogenic Cr(vi) in groundwater and surface water, often exceeding drinking water standards.^127^

In real conditions, it is often extremely difficult to clearly separate the contribution of industrial emissions and natural processes, especially in areas with complex geology and a history of technogenic impact.^118,128^ The situation is exacerbated by the fact that anthropogenic activity can not only add new Cr(vi) but also actively influence the mobilization and formation of geogenic Cr(vi). For instance, intensive irrigation in agriculture can leach accumulated natural Cr(vi) from the unsaturated zone and transport it to groundwater.^122,129^ Changes in groundwater exploitation regimes (pumping) affect hydrodynamics and can lead to water contact with previously inactive chromium-containing horizons.^130,131^ Finally, the creation of local anomalous geochemical conditions, for example, the formation of an alkaline environment in the zone of influence of landfills or thermal power plant ash dumps, can contribute to increased mobility and persistence of Cr(vi).^129,131^

Anthropogenic activity is the predominant cause of significant contamination: for example, a global analysis of 203 sites contaminated with Cr(vi) showed that 68.95% of them were due to human intervention.^1^ Industrial sources are capable of creating zones with extremely high chromium content: for instance, at contaminated industrial sites, Cr(vi) concentrations in the topsoil can reach 6100 mg kg^−1^, and in the water of aquifers beneath such sites – 2090 mg L^−1^.^117^ For instance, groundwater at a smelter site showed Cr(vi) concentrations of 162.9 mg L^−1^ and 234.5 mg L^−1^ in highly polluted regions,^132^ and near Chromite Ore Processing Residue (COPR) dumps in the Mexico the Cr(vi) content in groundwater reached 121 mg L^−1^.^114^

Geogenic sources, primarily associated with the weathering of ultrabasic and basic rocks, also contribute to the presence of Cr(vi) in the environment. Although peak concentrations from geogenic sources are often lower than those from anthropogenic ones, they can also significantly exceed drinking water standards. For example, in the groundwater of areas with nickel laterites in the Philippines, Cr(vi) concentrations reach 0.213 mg L^−1^,^133^ and in mine waters of the Sukinda Valley, India, where chromium is of geogenic origin from ultrabasic rocks, Cr(vi) concentrations can be as high as 4.25 mg L^−1^.^123^ Nevertheless, statistical data indicate a difference in contamination levels: at sites with high natural Cr(vi) content, its concentration in water did not exceed 0.2 mg L^−1^ in 75% of cases, whereas 56.43% of anthropogenically contaminated sites were characterized by Cr(vi) concentrations in water in a wider and often higher range of 0 to 10 mg L^−1^.

### 3.2. Migration pathways and transformation of Cr(vi) in geospheres

Having entered the environment hexavalent chromium undergoes a complex cycle of migration and chemical transformations that determine its ecological fate. A fundamental property causing its widespread distribution is the high solubility of the chromate anion, which contrasts sharply with trivalent chromium (Cr(iii)) that forms poorly soluble hydroxides or binds firmly to solid phases.^33^

The key process controlling chromium toxicity and mobility in the environment is redox reactions. On one hand, reduction of Cr(vi) to Cr(iii) represents a crucial mechanism of natural attenuation, converting chromium to a less toxic and significantly less mobile form. The main natural reducing agents include divalent iron (Fe(ii)),^7,33,134^ various forms of organic matter,^18,119,121^ and microorganisms capable of using Cr(vi) as an electron acceptor.^109,135^ On the other hand, the reverse process – oxidation of Cr(iii) to Cr(vi) – can regenerate the toxic form and maintain contamination. Since Cr(iii) exists as sparingly soluble forms while Cr(vi) is highly mobile at natural pH, Cr(iii) oxidation is prerequisite for chromium enrichment in groundwater.^136^ This oxidation begins with Cr(iii) release from its mineral matrix through weathering processes. Even minerals with relatively low chromium concentrations compared to chromite (*e.g.*, chlorites, pyroxenes) can be significant sources due to their greater weathering susceptibility.^137^

The primary natural oxidizers of Cr(iii) are high-valent manganese oxides (MnOx), particularly mixed-valence Mn(iv/iii)-oxides.^5,125,137,138^ The oxidation mechanism involves Cr(iii) adsorption onto Mn-oxide surfaces, followed by electron transfer leading to Cr(vi) formation and manganese reduction.^137^ Importantly, Mn oxides can promote chromite oxidative dissolution even under anoxic conditions, as demonstrated in basalt-origin soils where high Cr(vi) levels occurred in horizons with co-existing Cr(iii)-minerals and Mn(iii/iv) oxides.^139^ However, recent studies have revealed alternative oxidation pathways. Dissolved oxygen (DO), typically present at 1.7–6.39 mg L^−1^ in high-Cr groundwater,^136^ contributes both directly and indirectly to oxidation. In sedimentary aquifers, both Mn oxide-mediated and DO-mediated oxidation during silicate weathering generate Cr(vi), particularly with long residence times.^136^ DO also oxidizes dissolved Mn(ii) to Mn-oxides, which then act as chromium oxidants.^137^

Interestingly, in ophiolitic aquifers, trivalent iron (Fe(iii)) present in serpentinites has been identified as a primary oxidant,^140^ with the Fe_2_O_3_/(FeO + Fe_2_O_3_) ratio in serpentine controlling Cr concentrations over considerable dissolution extents.^140^ Additionally, hydrogen peroxide (H_2_O_2_) can serve as an important oxidant in ultramafic environments, even under anaerobic conditions.^137^ Microbial activities also significantly influence the process, with Mn(ii)-oxidizing fungi showing varying effects: Cr(iii) promotes hyphae-mediated Mn(ii) oxidation but inhibits enzyme-mediated processes.^141^ The oxidation rate and extent depend on Cr(iii) speciation, fungal Cr(vi) removal capacity, and organic content.^141^

The direction of chromium redox transformations is controlled by geochemical conditions, primarily redox potential (*E*_h_) and pH.^138,142^ Cr(vi) prevails at *E*_h_ values above 450–550 mV, while Cr(iii) dominates below this threshold.^140^ The influence of pH is complex: surface-catalyzed Mn(ii) oxidation peaks at pH 9,^137^ while Cr(vi) stability maximizes under alkaline conditions.^17,129^ At typical groundwater pH (6.5–8.5), Cr(iii) solubility remains low (<5 μg L^−1^), favoring the mobile Cr(vi) form.^125^ Environmental factors also play crucial roles. Organic matter has dual effects: enhancing Cr(iii) release from minerals while potentially inhibiting oxidation through stable complex formation.^137^ Agricultural activities promote oxidation through ammonium fertilizer-induced acidification and phosphate fertilizer-enhanced chromate desorption.^137^ Since ultramafic environments show inherent Cr(iii) oxidation capacity, retention processes ultimately determine Cr(vi) contamination extent.^143^

Beyond redox transformations, sorption and desorption at water–solid interfaces critically control Cr(vi) migration. Chromate anions interact with iron/aluminum oxides and clay minerals through electrostatic attraction and surface complexation.^18^ However, this binding strongly depends on pH: sorption maximizes below pH 6–7 but weakens dramatically above pH 7–8 as mineral surfaces become less positive or negative.^5,17,18^ Competing anions, particularly phosphate and sulfate, can reduce sorption effectiveness and cause chromate desorption.^128,144^ These reversible processes provide only temporary retention, and subsequent environmental changes can remobilize chromium.^145^

Physical transport mechanisms further influence chromium migration. Advection with groundwater flow represents the primary transport mechanism, with Cr(vi) migration rates potentially reaching meters to tens of meters annually.^33^ Hydrodynamic dispersion gradually expands contamination plumes,^130^ while hydrogeological properties significantly affect migration patterns. Low-permeability layers can slow vertical migration and create Cr(vi) accumulation zones,^117,119^ and low flow velocities in sedimentary aquifers promote Cr(vi) generation and accumulation.^125,136^

The observed chromium behavior at any location results from the complex interplay of these processes: oxidation–reduction, sorption–desorption, and physical transport. This dynamic balance is highly sensitive to site-specific geochemical conditions (pH, mineralogy, organic content) and hydrogeological factors (flow rates, porosity, saturation). The multifactorial nature of these interconnected processes makes accurate prediction of Cr(vi) environmental fate particularly challenging.

### 3.3. Main accumulation zones of hexavalent chromium

The widespread distribution and persistent accumulation of Cr(vi) in the environment, driven by its migration and transformation, is a significant ecological problem. Its mobility and stability lead to accumulation in natural reservoirs, creating long-term contamination hotspots. These pose substantial risks to ecosystems and human health, demanding comprehensive attention to the numerous and diverse sources of this pervasive pollutant.

Groundwater contamination is of greatest concern, as Cr(vi) actively migrates into this vital resource from a wide spectrum of industrial and geogenic sources,^1^ facilitated by the high solubility and mobility of chromate ions. Studies worldwide show Cr(vi) distribution in diverse aquifers, from shallow^146^ to deep.^131^ Surface waters (rivers, lakes, wetlands) are also contaminated through direct discharges, runoff, or groundwater discharge;^120,134^ while dilution can occur in large bodies, local levels can be significant. Atmospheric transport of Cr(vi) in aerosols,^147^ especially near industrial or combustion zones, contributes to wider geographical spread through particle precipitation. Furthermore, chromium can enter biological cycles; plants absorb it from soil and water,^135^ with accumulation varying by species and conditions, creating a potential pathway to food chains and humans.

Studies worldwide highlight its presence in pronounced industrial zones ^130,146^ and geologically specific regions.^5,7^ Industrial hotspots demonstrate the most extreme contamination. Extreme Cr(vi) levels (tens to hundreds of mg L^−1^) occur near COPR disposal or old chromate facilities.^113,114^ High concentrations (sometimes mg L^−1^) are also found near electroplating industries^121,146^ and metallurgical slag dumps.^111,112^ Intensive leather production^120,148^ causes complex wastewater and water body contamination. Mining^41,123^ and general urban/industrial pollution^149,150^ also contribute significantly. Alongside these, diverse geogenic sources, primarily weathering of Cr-containing rocks, contribute substantially. Ophiolite regions^7,126^ often show elevated groundwater Cr(vi) (tens of μg L^−1^, sometimes >50–100 μg L^−1^), exceeding standards. The problem is frequently exacerbated by a complex interplay of natural and anthropogenic factors, such as in Greece, where geogenic ophiolite contamination is augmented by industrial sources like ash dumps.^115,118^ In California, geogenic factors are worsened by anthropogenic impacts like irrigation-induced mobilization.^122,129^ China shows a full spectrum: industrial site contamination,^119,130,131^ agricultural impacts, natural Cr(vi) in deep aquifers,^127,145^ and atmospheric Cr.^149^

**Fig. 3:**
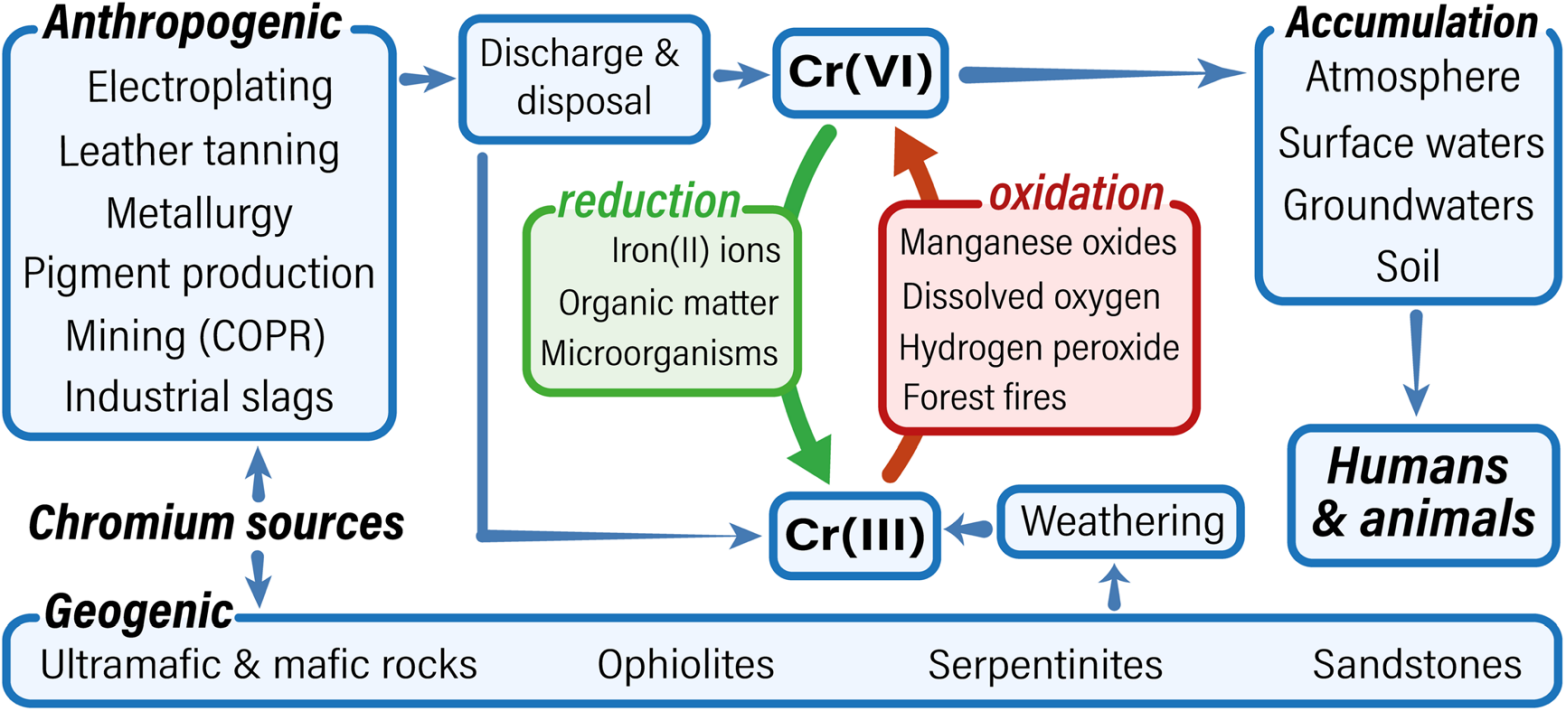
Scheme of chromium contamination pathway.

Global Cr(vi) contamination, stemming from multiple sources and complex migration pathways, reflects diverse regional factors and poses profound systemic risks. This environmental cycle is visually summarized in Fig. 3. Considering chromium's wide distribution, stability, complex geochemistry, limited natural attenuation, and critically, its numerous and diverse sources of contamination, these challenges demand stringent control over all sources throughout its cycle. This also necessitates developing and applying effective, though often costly and long-term, cleanup measures to prevent further catastrophic contamination and protect ecological and human health.

## 4 Modern approaches to chromium(vi) determination in aqueous media

The scale of environmental contamination with chromium compounds requires the development of mass, rapid, and accessible monitoring methods capable of providing operational control over large territories and in real-time. Traditional instrumental analytical methods, such as inductively coupled plasma mass spectrometry (ICP-MS),^151^ atomic absorption spectroscopy (AAS),^152^ and ion chromatography,^153^ while providing high accuracy and low detection limits, are not suitable for mass screening of chromium contamination. These methods require expensive stationary equipment, lengthy sample preparation, qualified personnel, and cannot be used for on-site analysis. In the context of monitoring vast territories and numerous sampling points, such limitations make traditional methods ineffective for operational contamination control.

The standards for chromium content in drinking water established by international organizations – 50 μg L^−1^ according to WHO recommendations^93^ and 100 μg L^−1^ according to EPA standards^95^ – serve as guidelines for researchers developing analytical methods. Therefore, the vast majority of developed sensor systems demonstrate detection limits significantly below these regulatory values,^27,154–156^ making method comparison based solely on this parameter uninformative. Considering the severe toxicological risks and the vast scale of environmental contamination detailed in the preceding chapters, the demands for effective monitoring take on special significance. For large-scale control, the key characteristics are no longer just laboratory accuracy, but rather portability, selectivity, rapid analysis time, and the capability for on-site field operation.

Colorimetric methods remain the simplest to implement in portable devices, as color change can be easily registered using a photodiode or smartphone camera.^157,158^ The classical method using diphenylcarbazide (DPC) continues to evolve through integration with modern materials.^27,159^ Immobilization of DPC on various supports allows creating ready-to-use test systems with long shelf life. An important advantage of colorimetric methods is their high selectivity – many systems demonstrate specific response to Cr(vi) even in the presence of high concentrations of other metal ions.^27,160^

Fluorescent methods provide higher sensitivity through light emission registration, which can be measured using compact photodiodes or portable fluorimeters.^29,161,162^ Carbon quantum dots of various nature work primarily through fluorescence quenching mechanism – Cr(vi) causes fluorescence intensity decrease due to inner filter effect or energy transfer. Some systems demonstrate more complex “on–off–on” behavior, where fluorescence is first quenched by Cr(vi) and then restored upon addition of reducing agents such as ascorbic acid.^161^ Such systems allow simultaneous determination of both Cr(vi) and reducing agents. Carbon quantum dots can also operate in dual mode – providing both fluorescent and colorimetric response, which increases analysis reliability.^163,164^ Metal–organic frameworks (MOFs) stand out among fluorescent materials due to their unique porous structure, which provides not only detection but also Cr(vi) preconcentration.^165–167^ Luminescent lanthanide-based MOFs demonstrate exceptional selectivity – they can detect Cr(vi) in the presence of multiple other ions without significant interference.^168,169^ An important advantage is the possibility of creating MOF-based solid-phase sensors in the form of films or test strips, simplifying their practical application.^170,171^

Electrochemical methods differ in their miniaturization potential. Potentiometric sensors with ion-selective membranes can be easily miniaturized to the size of portable pH meters.^172–174^ Meanwhile, methods based on anodic stripping voltammetry require more complex equipment for generating potential sweeps and recording voltammograms, although modern screen-printed electrodes and portable potentiostats have significantly simplified their field application.^175–177^ Electrochemical methods provide high selectivity through selection of Cr(vi) reduction potential, minimizing the influence of other electroactive substances.^178,179^

Photoelectrochemical sensors register photocurrent generated upon illumination of semiconductor material, requiring a light source and simple current measurement circuit.^180–182^ Using sunlight as excitation source makes such sensors particularly attractive for field measurements.^183,184^ While selectivity is intended to arise from the specific interaction of Cr(vi) with photogenerated charge carriers, it may be a significant practical limitation. The total photocurrent signal is susceptible to interference from any other species in a sample that can also react with the electrons or holes.

Microfluidic paper-based devices (μ-PADs) represent a unique platform combining advantages of various detection methods with the simplicity of paper test strips.^185,186^ Capillary forces provide liquid transport without external pumps, and integration of preconcentration methods allows achieving low detection limits.^187^

The developed sensor systems are successfully applied for analyzing various sample types. Natural and wastewater feature relatively simple matrices, allowing direct measurements.^183,188^ Soil analysis requires preliminary extraction, for which special field protocols using alkaline solutions have been developed.^189–191^ For food products, accounting for matrix effects and possible interferences from organic components is important.^192,193^

Integration of sensors with smartphones and portable devices opens new possibilities for mass monitoring.^28,158,194^ Using machine learning algorithms allows improving analysis accuracy and compensating for measurement condition variations.^157,195^ Creating sensor networks with real-time data transmission enables contamination mapping and spread prediction.^30,196^

An important trend is the development of multifunctional materials capable of not only detecting but also removing or neutralizing Cr(vi).^197,198^ Such systems are particularly promising for creating integrated monitoring and remediation devices for contaminated waters, aligning with modern concepts of sustainable development and circular economy.

## 5 The search for effective methods of Cr(vi) detoxification

**Fig. 4:**
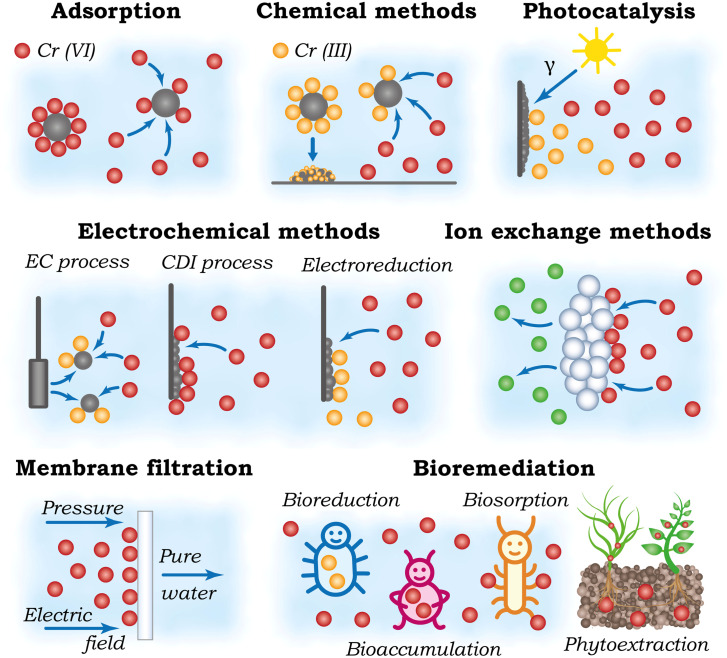
Schematic representation of the operating principles of main technologies investigated for hexavalent chromium removal from water.

The significant toxicological risks and widespread environmental presence of hexavalent chromium (Cr(vi)) have necessitated the development of various removal technologies. This chapter provides a systematic overview of the primary remediation strategies investigated, including adsorption, chemical reduction, electrochemical methods, photocatalysis, ion exchange, membrane filtration, and bioremediation, with their operating principles schematically summarized in Fig. 4. While laboratory studies for these technologies consistently report high removal efficiencies, a critical evaluation is required to assess their practical viability. The chapter, therefore, concludes with a comparative analysis of these methods, evaluating their operational limitations, economic factors, and overall applicability to real-world contamination scenarios to identify the barriers impeding their large-scale implementation.

### 5.1. Adsorption techniques for Cr(vi). Scope and constraints

Adsorption represents an effective method for Cr(vi) removal from aqueous solutions through surface concentration of chromate and dichromate anions at the solid–liquid interface. The process efficiency depends critically on adsorbent surface chemistry and solution pH, with maximum adsorption typically observed under acidic conditions where positively charged surfaces (from protonated functional groups) electrostatically attract Cr(vi) anions. Beyond electrostatic interactions, specific adsorption occurs through coordination or hydrogen bonding with surface functional groups, while certain adsorbents containing reducing components (Fe(ii), sulfide groups) can simultaneously reduce toxic Cr(vi) to less harmful Cr(iii).^199^ Adsorbent performance is characterized by adsorption capacity (mg g^−1^), specific surface area (m^2^ g^−1^), and porosity, with adsorption behavior described through isotherms such as Langmuir and Freundlich models.^200^ Laboratory studies consistently demonstrate promising results with removal efficiencies often exceeding 95%.^201–203^ The reported maximum adsorption capacities vary dramatically across different material types. Natural sorbents and waste materials typically show moderate values, while specially synthesized nanostructured adsorbents achieve exceptionally high capacities. This wide range reflects the ongoing research drive to optimize performance while maintaining cost-effectiveness. Conditionally, successful laboratory adsorbents can be divided into several groups.

#### 5.1.1 Low-cost natural and waste materials

Their main advantage is availability and low cost. These include natural minerals,^204^ various biomass wastes,^205,206^ and industrial wastes.^202^ Many of them show decent adsorption capacity in laboratory tests, sometimes reaching tens or even more than a hundred mg g^−1^ (for example, 117 mg g^−1^ for pistachio shells^206^), positioning themselves as promising “green” sorbents.

#### 5.1.2 Treated and activated materials

This group includes materials obtained from natural raw materials or wastes through special treatment (for example, acid, alkaline, thermal activation) to improve their sorption properties. Classic examples are activated carbons^207,208^ and biochars,^207,209^ which, due to developed porous structure and large specific surface area, often demonstrate increased adsorption capacity compared to the original raw materials (for example, 103 mg g^−1^ for alkali-treated roots of harpagophytum procumbens *versus* 77.24 mg g^−1^ for untreated roots ^210^).

#### 5.1.3 Synthetic and nanostructured materials

This most extensive and actively developing group includes materials created by targeted synthesis to achieve maximum efficiency. Here figure nanoparticles of metal oxides,^211,212^ various carbon nanomaterials (CNTs, graphene and its oxide, nanofibers),^201,212^ metal–organic (MOF)^213^ and covalent organic (COF) frameworks,^19^ as well as various composites, often combining nanoparticles with polymer or carbon matrix.^200,214,215^ For many such materials, very high adsorption capacity values are claimed (hundreds^207,215,216^ and even thousands^214^ mg g^−1^), rapid adsorption kinetics, and the possibility of imparting additional useful properties, for example, magnetic for easy adsorbent separation.^201,217^

Additionally, for many adsorbents from different groups, the possibility of regeneration and reuse in several cycles with retention of a significant part of the initial efficiency is demonstrated in laboratory conditions.^203,207,211^ However, the practical implementation of adsorbent regeneration can be a labor-intensive and costly process. Consequently, there is growing interest in cost-effective, single-use applications, which particularly elevates the importance of “green adsorbents” derived from readily available or waste resources.^218^ In this vein, materials such as Syzygium cumini bark biosorbents have demonstrated exceptional efficacy (adsorption capacity over 500 mg g^−1^, Freundlich model) in treating tannery wastewater with high Cr(vi) concentrations.^219^ Materials derived from agricultural waste, such as biochar from rice husks modified with polyethylenimine and KOH (PEI–KOH alkali-rice husk derived biochar^220^), demonstrate impressive results, exhibiting a Langmuir adsorption capacity of up to 435.7 mg g^−1^ for Cr(vi). Furthermore, biosorbents from fruit waste, like mango kernel bio-composites, have also shown remarkable performance with Langmuir adsorption capacities reaching 322.58 mg g^−1^ for Cr(vi), underscoring the potential of minimally processed natural materials.^221^

### 5.2. Treating Cr(vi) *via* chemical pathways

Chemical treatment methods for Cr(vi) removal operate by converting highly soluble and toxic Cr(vi) into less toxic and significantly less soluble Cr(iii) through chemical reduction, followed by precipitation or coagulation to form easily separable solid phases. The reduction process involves adding electron-donating reducing agents such as sulfur-based compounds (sulfites) or iron-based materials (ferrous salts, metallic iron, including nanoscale forms) to convert Cr(vi) to the +3 oxidation state.^222–225^ Following successful reduction, Cr(iii) removal is achieved through precipitation and coagulation processes. Since chromium(iii) hydroxide (Cr(OH)_3_) exhibits very low solubility at neutral and slightly alkaline pH, simple pH adjustment can induce precipitation as a solid phase.^22^ Coagulation efficiency is enhanced by adding iron(iii) or aluminum salts, which form voluminous hydroxide flocs (Fe(OH)_3_, Al(OH)_3_) that capture Cr(OH)_3_ particles and facilitate settling.^22^ These processes are combined for optimal efficiency – iron(ii) sulfate, for example, simultaneously reduces Cr(vi) and precipitates both Cr(iii) and formed iron(iii) as mixed hydroxide precipitates.^22^

Laboratory and pilot-scale studies demonstrate significant potential for deep purification, consistently achieving Cr(vi) removal efficiencies exceeding 99% using various reducing agents under optimized precipitation and coagulation conditions.^22,225,226^ Zero-valent iron (ZVI), both as nanoparticles (nZVI) and conventional iron scrap, attracts considerable attention due to iron availability and superior reducing capacity compared to iron salts. Pilot studies using iron scrap in flow columns with subsequent hydroxide precipitation achieved 98.5% total chromium removal at optimal pH 7.6–8.0.^22^ Classical reducing agents including iron(ii) sulfate and sulfites (sodium metabisulfite) regularly demonstrate effectiveness in controlled conditions.^227^ Their combination with coagulation using iron(iii) or aluminum salts reduces total chromium concentrations below drinking water standards (<0.05 mg L^−1^) with >99% removal efficiency under optimized pilot condition.^22^ Combined reduction–coagulation–flocculation systems using modern polymer flocculants and specialized reactors achieve >97% Cr(vi) removal.^228^ Other promising reducing agents include polysulfides, which demonstrated high efficiency (<0.05 mg per L Cr(vi)) in model *in situ* aquifer treatment experiments,^229^ and the Fenton process (Fe^2+^/H_2_O_2_), which achieved 92% Cr(vi) removal through combined reduction and co-precipitation mechanisms.^230^

### 5.3. Electrochemical approaches to Cr(vi) remediation

Electrochemical methods utilize electrical energy to drive chemical and physicochemical processes for Cr(vi) removal or transformation in aqueous solutions through electrode systems comprising anodes and cathodes with applied current or voltage. The primary approaches include electrocoagulation, direct electrochemical reduction, and capacitive deionization (electrosorption).

Electrocoagulation (EC) generates coagulants *in situ* through electrochemical dissolution of sacrificial anodes (typically iron or aluminum), releasing Fe^2+^/Fe^3+^ or Al^3+^ ions that immediately hydrolyze to form highly active hydroxide flocs (Fe(OH)_3_, Al(OH)_3_).^231,232^ These flocs effectively bind and precipitate chromium, predominantly as Cr(iii) formed through reduction by Fe^2+^ ions, while also partially adsorbing Cr(vi).^233,234^ This approach offers superior process control since coagulant generation rates are regulated by current intensity.

Direct electrochemical reduction facilitates electron transfer from cathode surfaces to Cr(vi) ions, reducing them to Cr(iii).^235,236^ Process effectiveness depends critically on cathode material, applied potential, and solution conditions. Indirect reduction may also occur through electrochemically generated reducing agents in solution.^235^ The resulting Cr(iii) can be precipitated due to local pH increases at the cathode.^237^

Capacitive deionization (CDI) or electrosorption employs porous electrodes with high specific surface areas, often based on activated carbon materials.^238^ Applied voltage causes ions, including chromate anions, to migrate toward oppositely charged electrodes and become temporarily retained in the electric double layer (EDL) at the electrode-solution interface.^238^ Unlike conventional adsorption, CDI uses electric potential rather than physicochemical affinity as the driving force. A key advantage is process reversibility – removing or inverting voltage causes accumulated ions to desorb, enabling chemical-free electrode regeneration.

Laboratory and pilot studies demonstrate significant potential for effective and controlled Cr(vi) removal with reduced chemical loading compared to traditional reagent methods. Electrocoagulation has received extensive investigation, with numerous studies reporting very high chromium removal efficiencies. Treatment of real tannery effluents using Al/Ti electrodes with response surface methodology optimization achieved 99.58% Cr(vi) removal.^232^ Pilot groundwater treatment tests showed EC performance equivalent to chemical coagulation, achieving >99% total chromium removal and meeting drinking water standards.^22^ Advanced systems include EC with novel electrode materials such as porous NiO/NF, demonstrating 99.5% Cr(vi) removal in 20 minutes while generating hydrogen,^23^ and hybrid electrocoagulation–capacitive deionization (CDEC) systems reaching standards (<0.05 mg L^−1^) with claimed energy savings.^233^

Direct electrochemical reduction and electrosorption methods show promising laboratory-scale results. Studies demonstrate effective Cr(vi) reduction to Cr(iii) on various cathode materials,^235^ with optimized conditions (voltage, temperature, mixing) achieving high extraction rates (87–91% for copper/carbon electrodes) and potential energy savings.^236^ Capacitive deionization technologies offer reagent-free ion removal with potential selectivity advantages. Novel electrode materials including nanodiamond-modified carbon fabrics^237^ and composites based on layered double hydroxides and polypyrrole (NiFe-LDH/PPy)^238^ achieve high specific chromium capacities (up to 39.5 μmol g^−1^ (ref. 237) or 47.95 mg g^−1^ with theoretical maximum 111 mg g^−1^ (ref. 238)) and removal rates up to 95.9% for 100 mg per L Cr(vi).^238^ Flow-configuration CDI (FCDI) also demonstrates potential for selective Cr(vi) extraction from mixed solutions.^239^

### 5.4. Photocatalytic reduction of hexavalent chromium

Photocatalysis employs light energy (ultraviolet or visible radiation) to initiate and accelerate chemical reactions on semiconductor photocatalyst surfaces, with the primary objective of reducing toxic hexavalent chromium to its less toxic and less mobile trivalent form (Cr(iii)) in water treatment applications.^240–242^

The operational principle relies on semiconductor properties where photocatalyst absorption of light quanta with energy sufficient to overcome the band gap generates charge carrier pairs: electrons (e^−^) transition from the valence band to the conduction band, leaving positively charged holes (h^+^) in the valence band.^243^ These photogenerated electrons and holes initiate target reactions. Conduction band electrons possess sufficient reducing potential to directly reduce Cr(vi) ions adsorbed on catalyst surfaces to Cr(iii), representing the primary pathway for Cr(vi) removal in photocatalysis.^244,245^ Conversely, holes function as strong oxidizers that can react with water molecules or hydroxide ions to form highly reactive hydroxyl radicals (OH˙), or directly oxidize other substances present in water, including organic pollutants.^246^ The potential for utilizing solar energy makes this approach particularly attractive, driving extensive research toward developing materials that effectively operate in the visible spectrum rather than traditional UV-active catalysts like TiO_2_.^240,241^

Diverse visible light photocatalysts are actively developed and investigated, including individual materials and, particularly important for efficiency enhancement, composites and heterostructures. Studied systems encompass modified oxides (N–TiO_2_ (ref. 244) or Ag/WO_3_ as part of Ag/WO_3_/rGO composite^247^), various sulfides often acting as heterostructure components (CdS in CdS/LDH,^248^ Bi_2_S_3_ in BiOI/Bi_2_S_3_,^249^ SnS_2_ in SnS_2_/GO,^250^ and complex systems like Fe_3_O_4_@rGO@CdS/Bi_2_S_3_ (ref. 251)), graphitic carbon nitride (g-C_3_N_4_) including substrate-supported forms,^252^ metal–organic frameworks (MOF),^253^ covalent organic frameworks (COF),^19^ and MXenes.^24,242^ The primary objective in creating composites and heterostructures extends beyond improving light absorption to mainly increasing photogenerated electron–hole separation efficiency. This prevents rapid recombination and enables more electrons to participate in Cr(vi) reduction.

Laboratory conditions demonstrate high rates and degrees of Cr(vi) reduction for many photocatalytic systems. Reports frequently indicate removal (reduction) exceeding 90–98% of Cr(vi) within relatively short timeframes ranging from tens of minutes to several hours.^247–249,251,254^ Particularly impressive results are achieved with certain heterostructures where nearly complete reduction occurs within 10–40 minutes of visible light irradiation.^249^ Successful photocatalytic applications are demonstrated not only in model solutions but also in treating real industrial effluents.^247^ Multifunctional systems are being developed that combine photoreduction capabilities with adsorption, detection, and subsequent chromium processing functions.^214^

### 5.5. Ion exchange resins for Cr(vi) removal

Ion exchange represents a reversible and stoichiometric chemical process where unwanted dissolved ions are exchanged for other ions initially bound to solid insoluble materials called ion exchangers or resins, with exchange occurring in equivalent quantities.^255^ Ion exchangers typically consist of synthetic polymeric resins in granular^256^ or fiber^257^ forms, containing fixed ionic groups and mobile counterions capable of exchange.

Since hexavalent chromium exists in water as anions, anion exchange resins (anionites) are employed for its removal.^258,259^ These resins contain positively charged functional groups (such as quaternary ammonium groups in strongly basic anionites) firmly fixed on polymer matrices, with mobile anions (typically chloride or hydroxide ions) compensating this charge. When contaminated water passes through resin layers (usually in column apparatus), an exchange process occurs where negatively charged Cr(vi) anions from solution displace original mobile anions (*e.g.*, Cl^−^) from resin active sites and bind to the positively charged matrix. Displaced ions transition into the purified water while toxic chromium becomes concentrated in the ion exchanger phase.

When significant portions of resin exchange sites become occupied by (bi)chromate anions (resin “exhaustion”), capacity decreases and regeneration becomes necessary. This is achieved by passing concentrated reagent solutions (NaCl or NaOH) containing high concentrations of original or competing anions through the resin. These anions displace accumulated chromium back into solution as concentrated eluate, restoring the resin to its original form.

Scientific literature presents ion exchange as a mature and potentially highly effective technology for ionic pollutant removal, including Cr(vi). Laboratory and column experiments using Amberlite IRA400 resin for real electroplating effluent purification achieved up to 96.7% Cr(vi) removal.^260^ Commercial Indion GS-300 resin under optimized conditions demonstrated high adsorption capacity (294 mg g^−1^) and 98.2% removal efficiency.^259^ New materials are actively developed including acrylic anion exchange fibers^257^ and modified structure resins^20^ showing high capacity and efficiency, plus hybrid systems (NZVI-resin) promising for final drinking water purification from trace Cr(vi) amounts.^224^

The key advantage emphasized in numerous studies is the regeneration capability and repeated use of ion exchange materials. Laboratory conditions demonstrate successful completion of multiple adsorption–regeneration cycles for various resins, including 30 cycles for Amberlite IRA400 maintaining >97% efficiency^260^ and 5 cycles for PADD fiber maintaining >95% capacity.^257^ This regeneration possibility offers prospects for reduced operating costs and decreased solid waste generation compared to non-regenerable adsorbents.

### 5.6. Membrane based solutions for Cr(vi)

Membrane technologies comprise separation processes utilizing semipermeable membranes – thin barriers capable of passing certain mixture components (typically water) while retaining others (dissolved substances, including Cr(vi) ions). Process driving forces include pressure differences, electrical potentials, or concentration gradients. All these technologies employ membranes as key selective elements separating initial flows into purified water (permeate) and concentrated waste streams (concentrate or retentate).^261^

The main methods include nanofiltration (NF) and reverse osmosis (RO), which are baromembrane processes where high pressure forces water through membranes that retain Cr(vi) ions through sieving effects and electrostatic interactions.^21,262,263^ Studies demonstrate that NF membranes effectively retain multivalent ions, achieving 95–96.5% Cr(vi) rejection rates for commercial membranes treating industrial effluents,^21^ while RO membranes with denser structures provide nearly complete chromium and salt removal with rejection rates exceeding 99%.^264^ Efficiency can be enhanced through advanced approaches such as micelle-enhanced nanofiltration (MENF), achieving up to 98.5% Cr(vi) removal.^262^ Electrodialysis (ED) employs a different principle where electric fields cause Cr(vi) ions to migrate through selective anion exchange membranes toward anodes, concentrating them in separate streams.^265^ ED demonstrates potential for selective Cr(vi) transfer^266^ and is studied in combination with other methods including photocatalysis^244^ and CDI.^267^ Other membrane approaches under investigation include liquid membranes with selective carriers^267^ and hybrid processes using functionalized membranes (often nanofiber),^215,268–271^ which can combine filtration with surface adsorption or reaction.

Recent research has focused extensively on developing new membrane materials with improved or hybrid properties. Electrospun nanofiber membranes attract significant interest due to their high porosity, large specific surface areas, and functionalization possibilities.^271^ Studies report composite nanofiber membrane development, including polyacrylonitrile (PAN) with polyaniline (PANI) and polysulfide coatings^215^ or recycled PVC with cationic groups,^269^ which combine filtration with Cr(vi) adsorption and demonstrate high removal rates (>90%) in dynamic tests on model industrial effluents. These membranes also show good regeneration capability in laboratory conditions (10–12 cycles). Mixed matrix membranes incorporating functional fillers such as modified g-C_3_N_4_ (ref. 270) or boehmite nanoparticles with polyphenols^268^ into polymer bases (polysulfone or polyethersulfone) achieve very high Cr(vi) rejection rates (>92% and even >99%) while maintaining good water permeability. Liquid membranes in laboratory flow systems demonstrate high (>98%) and stable Cr(vi) removal efficiency when utilizing suitable carriers.^272^

### 5.7. Bioremediation strategies for hexavalent chromium

Biological treatment methods, or bioremediation, utilize the natural abilities of living organisms (microbes and plants) for Cr(vi) removal, detoxification, or stabilization in environmental systems.^273,274^ These approaches attract attention as potentially environmentally friendly and economical solutions operating through several key mechanisms. Biosorption represents a predominantly passive process where Cr(vi) or Cr(iii) ions bind to microbial cell surfaces (bacteria, fungi, algae) through physicochemical interactions with cell wall components, capsules, or extracellular polymeric substances without metabolic energy expenditure.^274^ Bioaccumulation involves active, energy-dependent processes where living cells absorb and accumulate chromium ions intracellularly through specialized membrane transport systems.^274^ Bioreduction is considered the most promising mechanism, as microorganisms use enzyme systems (chromate reductases) to reduce highly toxic Cr(vi) to less toxic and less mobile Cr(iii), which often precipitates as insoluble compounds.^274^ Phytoextraction employs plants' ability to absorb chromium through root systems and transport it to aboveground parts for accumulation, with hyperaccumulator plants capable of concentrating metals at high levels.^275^

Microbial remediation research focuses primarily on Cr(vi) bioreduction as the key detoxification mechanism. Studies report isolation of bacterial and fungal strains from contaminated environments with remarkable Cr(vi) resistance, surviving concentrations in thousands of mg L^−1^.^276^ Resistant strains from genera including *Bacillus*,^276–278^*Micrococcus*,^276^*Rhodobacter*,^25^ and *Trichoderma*^279^ demonstrate high bioreduction efficiency in laboratory conditions, often achieving 90–100% Cr(vi) to Cr(iii) conversion within hours or days under optimized conditions. Reported achievements include complete reduction of 100 mg per L Cr(vi) in 48 hours by bacterial isolates from tannery effluents^276^ and 92–98% Cr(vi) removal in bioreactors.^25,277,280^ Microalgae and cyanobacteria demonstrate chromium removal capabilities by accumulating it in biomass, potentially useful for biofuel production.^281,282^

Phytoremediation studies have identified hyperaccumulator plants capable of high-level chromium tissue accumulation, such as aquatic plant *Callitriche cophocarpa* accumulating up to 1274 mg kg^−1^.^26^ Particularly promising is the synergism between plants and microorganisms, where plant inoculation with specific endophytic bacteria or mycorrhizal fungi significantly increases chromium resistance, improves growth, and substantially enhances accumulation or removal efficiency. Combined use of *Trichoderma* and *Rhizomucor* fungi with King Grass increased plant Cr accumulation by 64% and total soil Cr removal by 34% compared to plants alone.^279^ Bacterial consortium inoculation of *Callitriche cophocarpa* significantly improved Cr(vi) phytoextraction and plant physiological status.^26^

Biological approaches demonstrate broad possibilities for Cr(vi) interaction including binding, accumulation, and detoxification through reduction in laboratory and limited field conditions. Their key advantage lies in fundamental biocompatibility, making them particularly attractive for *in situ* remediation of contaminated natural environments through introduction of adapted microbial cultures or specific plant species in contaminated zones.^283^

The scientific response to Cr(vi) contamination has produced a wide spectrum of remediation technologies, from established physicochemical methods to emerging biological and photocatalytic systems. These approaches are fundamentally distinct, involving either the physical transfer of chromium between phases or its chemical transformation into the less mobile Cr(iii) state *via* reduction. The primary features and operational principles of these varied approaches are summarized in Table 2.

**Table 2:** Overview of primary Cr(vi) remediation technologies

Method	Features and advantages	Key materials	References
Adsorption	Utilizes low-cost natural and waste materials. Achieves high adsorption capacities with synthetic materials. Allows for regeneration and reuse	Biomass, activated carbons, biochars, metal–organic frameworks (MOFs), nanocomposites	19, 199–221 and 290–293
Chemical reduction and precipitation	A simple and scalable technology with high removal efficiencies (>99% in pilot studies). Zero-valent iron (ZVI) is an available and effective reducing agent	Ferrous salts (*e.g.*, FeSO_4_), sulfites, zero-valent iron (ZVI), scrap iron	22, 199 and 222–230
Electrochemical methods	Provides a high degree of process control *via* current regulation. High removal efficiencies (>99% for EC) are demonstrated. Capacitive deionization offers reagent-free regeneration	Sacrificial anodes (Fe, Al) for electrocoagulation, porous carbon electrodes for CDI	23, 231–239 and 294
Photocatalysis	Utilizes light energy, including solar, offering a potentially sustainable approach. High reduction rates (>90–98%) are shown in laboratory settings	TiO_2_, g-C_3_N_4_, metal–organic frameworks (MOFs), MXenes	19, 24, 214, 240–245, 247–252 and 254
Ion exchange process	A reversible process that allows for multiple regeneration and reuse cycles while maintaining high efficiency. High capacity is shown with commercial resins	Anion exchange resins (*e.g.*, Amberlite IRA400), acrylic anion exchange fibers	20, 224 and 256–260
Membrane technologies	Achieves very high rejection rates, often exceeding 95% for nanofiltration (NF) and 99% for reverse osmosis (RO). Can be combined with other processes in hybrid membranes	NF and RO polymer membranes, electrospun nanofiber membranes, mixed matrix membranes	21, 215, 244, 261–263, 265, 267–272 and 295
Bioremediation	A potentially eco-friendly and economical solution. Bioreduction provides a promising detoxification pathway. Can be applied for *in situ* treatment of contaminated sites	Bacteria, fungi, hyperaccumulator plants, microalgae	25, 26, 36, 273–283, 296 and 297

### 5.8. Comparative assessment of Cr(vi) remediation technologies

Comparative analysis of the reviewed chromium remediation methods reveals significant differences in their practical applicability and effectiveness. Removal efficiency varies substantially between methods and strongly depends on application conditions. Under laboratory conditions, many technologies demonstrate impressive results: adsorption on nanomaterials achieves 93–99.99% removal,^284^ electrocoagulation shows 95–100% efficiency,^285^ chemical precipitation provides 98–99% removal,^286^ and photocatalytic reduction achieves 95–100% within relatively short timeframes.^37^

However, it is worth noting that nearly all published remediation methods report such high removal efficiencies. This phenomenon is partly a result of publication bias, as high performance is often a prerequisite for acceptance in reputable journals. Consequently, removal efficiency as a percentage has become a less discerning metric for comparing different published technologies; a high value is practically a given. This shifts the focus to more critical, real-world criteria like cost, scalability, and overall practical applicability.

The percentage removal metric itself is also inherently biased. Achieving 99% removal is significantly easier when treating water with a high initial Cr(vi) concentration (*e.g.*, reducing it from 100 mg L^−1^ to 1 mg L^−1^) than when attempting to remove trace amounts to meet stringent regulatory standards (*e.g.*, from 0.1 mg L^−1^ to 0.001 mg L^−1^). The ultimate goal of any water treatment is to reach concentrations below these regulatory limits. It is precisely the removal of these final, trace concentrations that poses the greatest challenge, due to fundamental scientific barriers. Thermodynamically, the driving force for processes like adsorption or reaction diminishes as the concentration gradient between the solution and the material surface decreases. Kinetically, the probability of a successful collision between a chromium ion and an active site on the remediation agent becomes much lower at dilute concentrations. Therefore, a more rigorous assessment of any new technology should focus on its ability to reach specific target final concentrations from realistic initial levels, rather than on an often-deceptive removal percentage. This principle of realistic assessment must also extend to the testing environment. The high efficiencies reported in laboratories are achieved under idealized conditions, whereas real efficiency under industrial conditions may be significantly lower due to the influence of competing ions and complex wastewater composition.^287–289^

The critical dependence on pH represents a fundamental limitation for most methods. Adsorption typically requires acidic conditions (pH 2–4) for maximum efficiency,^291,292^ while real industrial effluents often have neutral or alkaline pH,^298^ necessitating costly pH adjustment steps. Similarly, ion exchange faces challenges from the chemical aggressiveness of chromate ions, which can oxidize and destroy both the polymer matrix and functional groups of resins.^258^ During multiple sorption–desorption cycles, Cr(vi) can be partially reduced to Cr(iii) on the resin,^20,257,258^ indicating undesirable side reactions and complicating regeneration. The presence of competing anions (phosphates, chlorides, nitrates) further reduces the practical efficiency of ion exchange by competing for active sites.^289^

Membrane technologies face serious fouling problems, where surfaces and pores become progressively clogged with suspended particles, colloids, organic substances, salt deposits, or biofilms.^295^ This leads to performance decline, increased energy consumption, and shortened membrane lifetime. Regular chemical cleaning with aggressive reagents is necessary but can damage membranes and generate additional waste streams requiring treatment.^295^ Furthermore, membrane processes merely redistribute pollutants rather than destroy them, generating highly concentrated waste streams that require additional treatment stages.

Cost parameters vary widely among technologies. Bioremediation and low-cost adsorbents from waste materials are positioned as the most economically accessible solutions.^36,299,300^ Chemical precipitation shows the lowest capital costs with potentially low operating expenses,^286^ though it generates large volumes of toxic chromium hydroxide sludge requiring landfill disposal.^286,301^ Electrochemical methods face limitations due to high electricity consumption and electrode replacement costs.^285^ The production cost of advanced nanomaterials can be 1000 times higher than activated carbon,^293^ severely limiting their practical application. For photocatalytic systems, while solar-driven processes offer minimal operational energy costs,^37^ the initial investment in reactor design and catalyst preparation, along with challenges in catalyst recovery and long-term stability, should impact economic viability.

Energy consumption represents a critical factor determining both economic feasibility and environmental sustainability. Biological methods, chemical precipitation, and adsorption require minimal electricity input.^36,284^ Solar-driven photocatalysis theoretically offers zero operational energy costs during daylight hours,^37^ though practical implementation faces challenges from light source variability and reactor design requirements. Electrochemical methods are inherently energy-intensive, with electrodialysis and electrocoagulation requiring continuous power input.^285,294^ Membrane processes, especially reverse osmosis, demand significant energy for pressure generation,^8^ making them among the most energy-consuming technologies.

The scalability of technologies from laboratory to industrial scale presents serious challenges. Most research on advanced adsorbents and photocatalysts has been conducted under idealized conditions using synthetic wastewater, with insufficient data on pilot or industrial-scale applications.^293^ The synthesis of complex nanostructures remains technically challenging and economically unfeasible for large-scale production. Biological methods face difficulties when scaling due to microorganism sensitivity to high pollutant concentrations and variable environmental conditions.^36,296^ The slow kinetics of biological processes, requiring weeks to months for significant effect, further limits their industrial applicability. Chemical methods and electrocoagulation demonstrate better scalability due to their relative simplicity and established industrial implementation.^285,299^

A fundamental problem inherent to all removal methods is the generation of secondary waste. Chromium, being a chemical element, cannot be destroyed but only transformed from one form to another or concentrated in different phases. Chemical precipitation generates large volumes of chromium hydroxide sludge,^286,301^ electrochemical methods produce metal-laden sludge and spent electrodes,^285,286^ membrane technologies create concentrated retentates requiring further treatment,^8^ and ion exchange produces toxic regeneration eluates.^39^ Even seemingly green approaches like adsorption on biomass or biological treatment ultimately generate chromium-laden solid waste requiring special disposal. This universal challenge highlights that Cr(vi) remediation merely transforms the contamination problem rather than solving it completely.

**Fig. 5:**
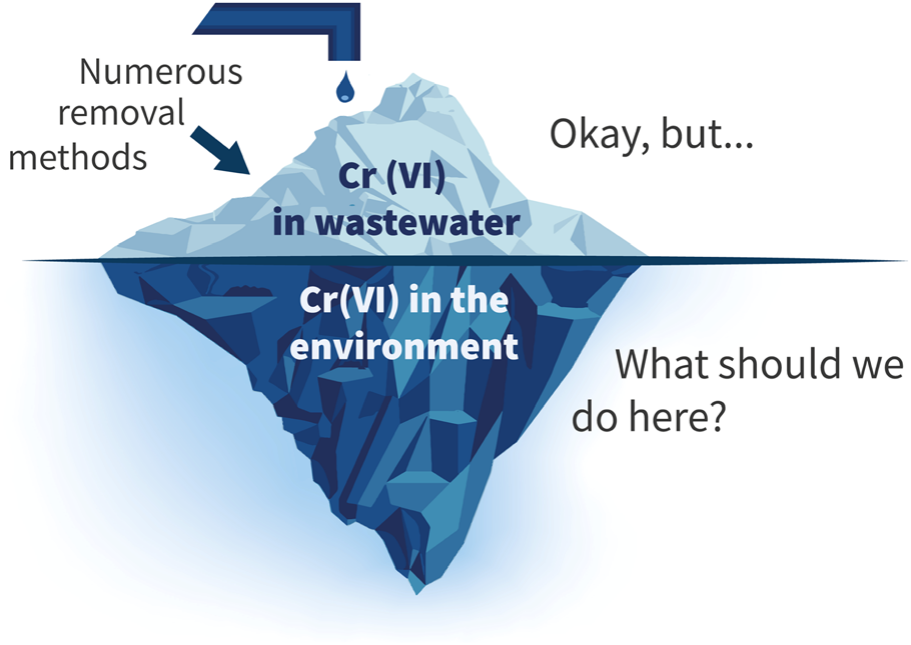
Metaphorical representation of the Cr(vi) contaminated sites problem.

The most critical limitation across all technologies, visually captured by the iceberg metaphor in Fig. 5, is their focus on treatment of wastewater – the small, visible tip of the problem – rather than addressing the vast scale of existing environmental contamination. Among reviewed methods, only bioremediation,^297^ nanoscale zero-valent iron injection,^302,303^ chemical stabilization/solidification,^301^ phytoremediation,^299,301^ and to some extent *in situ* photocatalytic treatment (in surface waters) can be applied directly at contamination sites. However, these *in situ* approaches face their own limitations: bioremediation suffers from slow kinetics and unpredictable performance, nZVI has limited reactive lifetime and potential for pollutant remobilization,^302^ phytoremediation is restricted to shallow contamination and low metal concentrations,^301^ while photocatalytic treatment requires adequate light penetration and catalyst stability.^37^

**Fig. 6:**
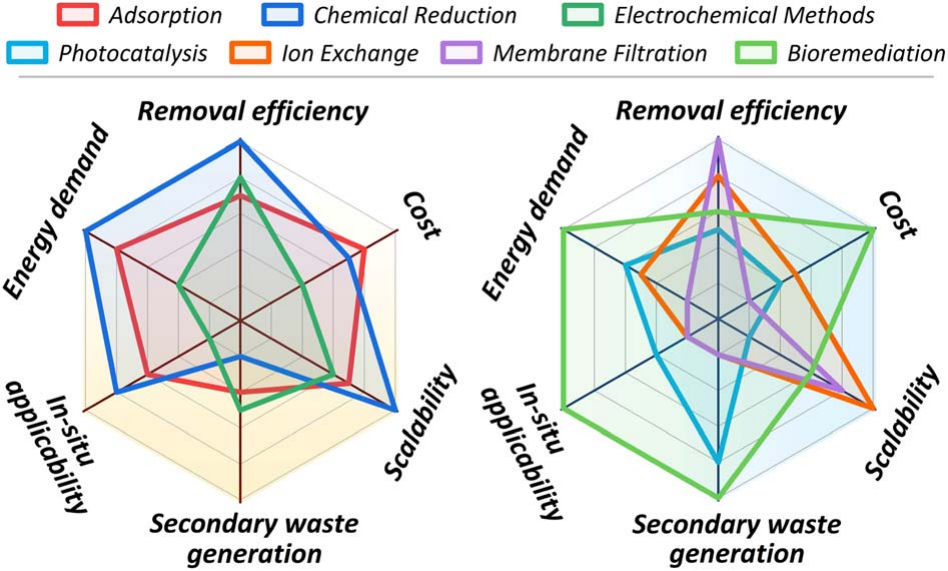
Comparative analysis of the main Cr(vi) remediation technologies using radar charts. The axes for cost, energy demand, and secondary waste generation are on an inverted scale, where a higher value represents lower value.

Considering environmental sustainability and the principles of green chemistry, the ideal remediation technology should minimize secondary waste generation, utilize renewable energy sources, avoid hazardous chemicals, and enable pollutant recovery or transformation into benign products. Based on these criteria, biological methods and solar-driven photocatalysis emerge as the most environmentally compatible approaches, despite their current limitations. Bioremediation offers the unique advantage of being a truly biocompatible process that can potentially restore damaged ecosystems, though its effectiveness remains unpredictable.^36,297^ Photocatalytic treatment, particularly when utilizing abundant materials like TiO_2_ and solar energy, represents a promising green technology,^37^ though challenges in scaling and real-world application persist. Hybrid approaches combining biological and photocatalytic processes, or integrating adsorption with photocatalytic regeneration, may offer synergistic benefits that overcome individual method limitations. A comparative visualization of these remediation technologies across performance indicators is presented in Fig. 6.

The development of truly sustainable Cr(vi) remediation necessitates a change in focus from simply transferring contamination between phases to creating technologies that can effectively immobilize chromium in geochemically stable forms or enable its recovery for reuse. This necessitates interdisciplinary collaboration between materials scientists, environmental engineers, and policymakers to develop economically viable solutions that address both immediate treatment needs and long-term environmental protection. Until such breakthrough technologies emerge, the selection of remediation methods will continue to involve compromises between efficiency, cost, and environmental impact, with no single technology providing a universal solution to the chromium contamination crisis.

## 6 Summary and prospects

This comprehensive review reveals a fundamental disconnect between our sophisticated understanding of hexavalent chromium toxicity and our limited ability to address environmental contamination. While research has elucidated molecular mechanisms of Cr(vi) toxicity with remarkable precision – from cellular entry through oxidative damage cascades to genomic instability – this knowledge has not translated into proportionally effective environmental solutions. The paradox extends beyond Cr(vi) itself: emerging evidence suggests that Cr(iii), traditionally considered safe, may possess significant biological activity when formed intracellularly or introduced as certain complexes. Even metallic chromium from medical implants presents underappreciated risks through continuous ion release. These findings challenge the fundamental assumption that reduction of Cr(vi) to Cr(iii) represents true detoxification, suggesting instead that current regulatory frameworks and remediation strategies may be built on incomplete toxicological understanding.

The scale of the problem becomes apparent when examining contamination patterns. While anthropogenic sources dominate both in frequency and concentration, the widespread occurrence of geogenic Cr(vi) adds complexity to attribution and management. Most critically, the inherent chemistry of chromium – particularly the reversibility of redox transformations between Cr(iii) and Cr(vi) under environmental conditions – means that contamination is not a static problem but a dynamic process. The presence of manganese oxides and other natural oxidants can regenerate toxic Cr(vi) from supposedly safe Cr(iii) deposits, creating persistent contamination cycles that current remediation approaches fail to address adequately.

Analysis of remediation practices reveals troubling feedback mechanisms that perpetuate rather than resolve contamination. Chemical precipitation, widely adopted for its economic feasibility, generates chromium-containing sludges requiring disposal. These disposal sites, particularly when containing manganese oxides or experiencing pH changes, can become secondary sources through re-oxidation of precipitated Cr(iii). Similarly, ion exchange resins accumulate chromium that must ultimately be managed, while membrane technologies concentrate pollutants without destroying them. Even biological treatment, often promoted as “green”, produces chromium-laden biomass requiring disposal.

The economic dynamics of chromium management create additional feedback loops. The high cost of advanced treatment technologies drives industries toward cheaper alternatives, often resulting in inadequate treatment or improper disposal. This is particularly evident in regions with weak regulatory enforcement, where short-term economic considerations override long-term environmental costs. Historical examples, such as chromite ore processing residue (COPR) disposal sites, demonstrate how decades of cost-effective practices create contamination legacies requiring orders of magnitude more resources to address than proper initial management would have required.

The proliferation of studies reporting near-perfect removal efficiencies under controlled laboratory conditions contrasts starkly with continued environmental contamination, suggesting that the limiting factor is not chemical feasibility but practical implementation. Most concerning is the tendency in materials science to develop complex nanocomposites or advanced materials first, then subsequently evaluate their application for various purposes including chromium removal. This approach, while producing impressive laboratory results, rarely considers scalability, cost-effectiveness, or real-world performance from the outset.

The focus on wastewater treatment, while important, has overshadowed equally critical challenges in soil and groundwater remediation. The vast majority of developed technologies target dissolved Cr(vi) in relatively controlled matrices, yet contaminated soils and aquifers represent the largest environmental reservoirs of chromium. The few technologies applicable to these matrices – primarily *in situ* chemical reduction or bioremediation – face significant limitations in heterogeneous subsurface environments. This gap between research focus and environmental need suggests that future development must prioritize technologies specifically designed for large-scale, *in situ* application rather than adapted from wastewater treatment approaches.

Recent advances in sensor technology offer transformative potential for chromium management, though this potential remains largely unrealized. The development of portable, rapid detection methods with sensitivity comparable to laboratory instruments enables continuous, distributed monitoring of contamination. Such capabilities could fundamentally change management strategies from periodic sampling to real-time surveillance, allowing early detection of contamination events and adaptive treatment responses. When integrated with data analytics and predictive modeling, these sensing networks could provide unprecedented understanding of chromium fate and transport in complex environments.

However, the true value of advanced monitoring extends beyond detection to enabling more nuanced management strategies. For contaminated sites where complete remediation is impractical, continuous monitoring could support risk-based management approaches that focus on preventing exposure rather than achieving arbitrary cleanup standards. This represents a pragmatic recognition that for many legacy contamination sites, perpetual management may be more realistic than remediation.

The evidence presented throughout this review points toward the necessity of fundamental shifts in how we approach chromium contamination. Rather than continuing to pursue increasingly sophisticated end-of-pipe solutions, emphasis must shift toward prevention and circular economy principles. This requires reimagining industrial processes to minimize chromium use, prevent Cr(iii) oxidation, and enable recovery and reuse rather than disposal. Where chromium use remains essential, closed-loop systems that maintain chromium in controlled chemical states throughout its lifecycle offer more promise than treatment of dispersed contamination.

For existing contamination, differentiated strategies based on contamination characteristics and site conditions are essential. High-concentration industrial releases may warrant aggressive chemical treatment despite secondary waste generation, while diffuse geogenic contamination might be better managed through stabilization and monitoring. Legacy contaminated sites, particularly those with deep subsurface contamination, may require acceptance of long-term containment and management rather than pursuit of complete remediation.

The chromium contamination crisis, as revealed through this comprehensive analysis, represents a more complex and persistent challenge than traditionally acknowledged. The combination of widespread historical contamination, ongoing industrial use, natural geogenic sources, reversible chemistry, and incomplete toxicological understanding creates a problem resistant to simple technical solutions. Moreover, the discovery that all chromium forms – including supposedly safe Cr(iii) and metallic chromium – may pose health risks under certain conditions necessitates fundamental reconsideration of management approaches.

Moving forward requires integration of multiple strategies rather than reliance on any single approach. Prevention must become the primary focus, with treatment reserved for situations where prevention fails. This demands close collaboration between researchers, industries, and regulators to develop practical, scalable solutions designed from inception for real-world implementation. Research priorities must shift from demonstrating theoretical possibilities to solving practical implementation challenges. The development of materials and technologies must be guided by clear understanding of deployment contexts, economic constraints, and lifecycle implications.

Perhaps most importantly, the scientific community must communicate honestly about the limitations of current approaches and the likely persistence of chromium contamination. Rather than perpetuating unrealistic expectations of complete remediation, effort should focus on developing robust, long-term management strategies that protect human health and ecosystems while acknowledging the practical constraints of chemistry, economics, and scale. Only through such realistic assessment and integrated action can we hope to minimize the ongoing impacts of this persistent environmental challenge.

## Author contributions

Yaroslav Zhigalenok: conceptualization, methodology, investigation, visualization, writing – original draft, writing – review & editing. Aigerim Tazhibayeva: validation, writing – original draft, writing – review & editing. Saule Kokhmetova: conceptualization, supervision, project administration, funding acquisition, writing – review & editing. Alena Starodubtseva: investigation, data curation, writing – review & editing. Tatyana Kan: investigation, resources, writing – review & editing. Dana Isbergenova: conceptualization, investigation, writing – review & editing. Fyodor Malchik: methodology, formal analysis, writing – review & editing.

## Conflicts of interest

There are no conflicts to declare.

## Data Availability

No primary research results, software or code have been included and no new data were generated or analysed as part of this review.
